# Reference equations for the six-minute walk distance in the healthy Chinese population aged 18–59 years

**DOI:** 10.1371/journal.pone.0184669

**Published:** 2017-09-14

**Authors:** He Zou, Xiuruo Zhu, Jia Zhang, Yi Wang, Xiaozhen Wu, Fang Liu, Xiaofeng Xie, Xiaoshu Chen

**Affiliations:** 1 Department of Cardiovascular Medicine, Wenzhou People’s Hospital, the Wenzhou Third Clinical Institute Affiliated with Wenzhou Medical University, Wenzhou, Zhe Jiang, China; 2 Department of Inspection Medical, Wenzhou People’s Hospital, the Wenzhou Third Clinical Institute Affiliated with Wenzhou Medical University, Wenzhou, Zhe Jiang, China; Forschungszentrum Borstel Leibniz-Zentrum fur Medizin und Biowissenschaften, GERMANY

## Abstract

**Background:**

The six-minute walk test (6MWT) is a safe, simple, inexpensive tool for evaluating the functional exercise capacity of patients with chronic respiratory disease. However, there is a lack of standard reference equations for the six-minute walk distance (6MWD) in the healthy Chinese population aged 18–59 years.

**Aims:**

The purposes of the present study were as follows: 1) to measure the anthropometric data and walking distance of a sample of healthy Chinese Han people aged 18–59 years; 2) to construct reference equations for the 6MWD; 3) to compare the measured 6MWD with previously published equations.

**Method:**

The anthropometric data, demographic information, lung function, and walking distance of Chinese adults aged 18–59 years were prospectively measured using a standardized protocol. We obtained verbal consent from all the subjects before the test, and the study design was approved by the ethics committee of Wenzhou People's Hospital. The 6MWT was performed twice, and the longer distance was used for further analysis.

**Results:**

A total of 643 subjects (319 females and 324 males) completed the 6MWT, and average walking distance was 601.6±55.51 m. The walking distance was compared between females and males (578±49.85 m vs. 623±52.53 m; p < 0.0001) and between physically active subjects and sedentary subjects (609.3±56.17 m vs. 592±53.23 m; p < 0.0001). Pearson’s correlation indicated that the 6MWD was significantly correlated with various demographic and the 6MWT variables, such as age, height, weight, body mass index (BMI), heart rate after the test and the difference in the heart rate before and after the test. Stepwise multiple regression analysis showed that age and height were independent predictors associated with the 6MWD. The reference equations from white, Canadian and Chilean populations tended to overestimate the walking distance in our subjects, while Brazilian and Arabian equations tended to underestimate the walking distance. There was no significant difference in the walking distance between Korean reference equations and the results of the current study.

**Conclusion:**

In summary, age and height were the most significant predictors of the 6MWD, and regression equations could explain approximately 34% and 28% of the distance variance in the female and male groups, respectively.

## Introduction

The six-minute walk test (6MWT) is a safe, simple, inexpensive tool for evaluating the functional exercise capacity of patients with chronic respiratory disease [1, 2]. Additionally, the six-minute walk distance (6MWD) is associated with death in some diseases [3–6]. Previously, some studies of healthy subjects found significant differences in the 6MWD [7–9]. Differences are commonly caused by differences in anthropometric factors, demographic factors, the racial background of the recruited subjects and the standardization methods applied in each study. In addition, the participants’ levels of daily physical activity, attitude towards the study and other factors may influence the 6MWD [9–13]. The American Thoracic Society (ATS) published the 6MWT guidelines and encouraged researchers to establish reference values for each population using these new guidelines [1]. However, there is a lack of standard reference equations for the 6MWD in the healthy Chinese population aged18-59 years.

The purposes of this study were as follows: 1) to measure anthropometric data and walking distance in a sample of healthy Chinese Han people aged 18–59 years according to the standardized means provided by the ATS guidelines; 2) to construct reference equations for the 6MWD; 3) to compare the 6MWD of our cohort with previously published equations.

## Methods

### Subjects

We collected data over a 40-month period from December 2013 to March 2017 from healthy subjects aged 18 to 59 years of age who were recruited to participate in this cross-sectional study. The study subjects came from a convenience sampling in Wenzhou City, which has an estimated population of over 9 million inhabitants. The subjects were medical personnel and workers from a public hospital, students and teachers at a local university and employees of two local private companies. Before the subjects were recruited for the study, they spoke with the researcher to understand the study’s purpose and to complete a questionnaire to ensure that all subjects were healthy. The researcher validated the questionnaire prior to administering it to study participants. We obtained verbal consent from all the subjects before the test, and that the study design was approved by the ethics committee of Wenzhou People's Hospital.

The followings were exclusion factors for our study:

- any problem with walking or requiring the use of walking aids.
- any organic disease.
- basic heart rate < 50bpm or ≥ 100 bpm.
- basic systolic blood pressure ≥ 150 mmHg or diastolic blood pressure ≥ 100 mmHg.
- respiratory symptoms for a month before the study.

### Physical activity questionnaires

Every participant was asked about the type, frequency, and duration of exercise activity for a month before the study. If the participants had performed lower limb exercises at least 20 minutes per session, 3 times per week in the last month, they were classified as “physically active” [14]. Participants who did not meet these criteria were classified as “sedentary”.

### Physical examination

The investigators measured the participants’ height, weight, and blood pressure and then calculated the BMI before the test. The participants’ ages were verified by their identity cards. Height was evaluated using a height gauge while the participants’ stood barefoot with a straight back. Weight (kg) was measured with an electronic scale, and the BMI was calculated for each subject as BMI = weight/height^2^ (kg/m^2^).

### Pulmonary assessment

Lung function was measured with a standard portable spirometer according to the ATS guidelines [15]. Forced expiratory volume in one second (FEV_1_), forced vital capacity (FVC) and FEV_1_/FVC were recorded before the test. Every participant needed to complete at least three pulmonary function tests; the largest value was recorded for analysis.

### Six-minute walk test

Two 6MWTs were completed according to the ATS guidelines[1]. The two 6MWTs were performed along a straight, long, flat, enclosed 30-meter corridor. Each 3 meters of the course was marked by an investigator. The turnaround points were marked with two special orange traffic cones. The starting line indicated the beginning and end of each 60 meters, which was marked with brightly colored tape on the floor. To minimize the effects of biological rhythms and temperature, the tests were completed between 9:00 a.m. and 1:00 p.m., and the temperature change ranged between 20°C and 25°C. Certain participant behaviors, such as strenuous exercise and eating a light meal within 2 hours of the start of the test, were forbidden. The participants needed to sit in a chair located near the starting line for at least 10 minutes before the start of the test. During that time, oxygen saturation (SpO_2_), resting heart rate and systolic and diastolic blood pressure before the test were recorded, and nurses calculated the maximum heart rate (mHR = 220—age). Each subject was informed that the aim of the test that was to see how far he or she could walk at his or her own pace in six minutes. Then, each subject was asked to walk up and down the corridor as fast as possible in six minutes. If subjects experienced dizziness, leg cramps, chest pain or dyspnoea, they were permitted to stop and rest. When the subjects were well again, they were encouraged to continue walking as soon as possible. The two 6MWTs were monitored by a single operator who recorded the walking distance at the same time. The operator needed to prompted the subjects every 60 seconds using standardized encouragement [1] (“you are doing well; you have five minutes left”, “good job; there are four minutes left”). The distance covered over the six minutes was recorded as the 6MWD. The nurses recorded the subjects’ oxygen saturation, systolic and diastolic blood pressure, and heart rate were recorded at the end of the test. Every participant was shown a modified Borg dyspnea scale [16] before beginning the test and at the end of the 6MWT; the scale of “0 = nothing at all” to “10 = very, very severe” was printed on a card, and the participants used the scale to assess their current degree of shortness of breath. The second test was completed two hours later.

The DOI link: http://dx.doi.org/10.17504/protocols.io. [i9sch6e].

## Data analysis

The main variables in the study had a normal distribution curve and were assessed with the Kolmogorov-Smirnov test; the data are presented as means with standard deviations (SDs). We assessed the associations between the 6MWD and the categorical variables (activity and gender) using the independent Student’s *t* test. Subject characteristics were assessed to determine their association with the 6WMD using a first univariate analysis with the Spearman’s correlation test and then added to the multivariate analysis according to the forward stepwise multiple linear regression results. A variables entry and removal from the model depended on whether the P-value was greater than 0.05, and collinearity with the multivariate analysis was detected using variance inflation factors. We compared the individual 6MWD with the distances predicted using other countries’ published equations for individuals with the same age ranges as in our study using the paired sample *t* test. Data analyses were performed using SPSS for Windows statistical software (version 15.0; SPSS, Inc., Chicago, IL). A p-value of < 0.05 was considered significant in all analyses.

## Results

### Demographic information, anthropometric data and lung function

In this study, a total of 707 healthy subjects (351 female and 356 male) were recruited from December 2013 to March 2017. Sixty-four subjects were excluded from the study, two because of a foot sprain, six because of a history of physician-diagnosed heart disease, twenty-nine because they had a basic heart rate < 50 bpm or ≥ 100 bpm or resting systolic blood pressure ≥ 140 mmHg or resting diastolic blood pressure ≥ 90 mmHg, twenty-five because they were underweight (BMI < 18 kg/m^2^) or overweight (BMI > 30 kg/m^2^), and five because they had respiratory symptoms. Finally, 643 subjects (319 females and 324 males) completed the 6MWT. No participant prematurely terminated the test or needed to rest during the test. The subjects’ characteristics are summarized in Table 1. The females were significantly shorter and lighter than the males, and there was a sex difference in BMI. More than half (56%) of the healthy subjects they performed lower extremity exercises for at least 20 minutes per session 3 times per week in the month before the study. All the subjects had normal lung function. The FEV_1_ values were higher than 80%, and the FEV_1_/FVC values were higher than 70% of the normal predicted value. There were significant differences in the FVC (L) and FEV_1_ (L) between the female and male subjects.

**Table 1:** Characteristics of the study subjects.

Characteristic	Females (n = 319)	Males (n = 324)	p-value*	Total (n = 643)
Age, years	40.0±12.04	39.5±12.37	NS	39.7±12.20
Height, cm	159.0±4.87	169.6±6.39	<0.001	164.3±7.76
Weight, kg	57.0±7.24	65.8±8.61	<0.001	61.4±9.08
BMI, kg/m2	22.6±2.91	22.9±2.70	NS	22.7±2.80
FEV_1_, L	2.5±0.36	3.4±0.59	<0.001	3.0±0.69
FVC, L	2.8±0.40	4.1±0.68	<0.001	3.5±0.84
FEV_1_/ FVC	0.87±0.02	0.84±0.02	NS	0.86±0.02

Values are expressed as the mean ± SD.

*p-value between males and females.

BMI: body mass index; FVC: forced vital capacity; FEV_1_: forced expiratory volume in one second.

### Six-minute walk distance

**Fig 1:**
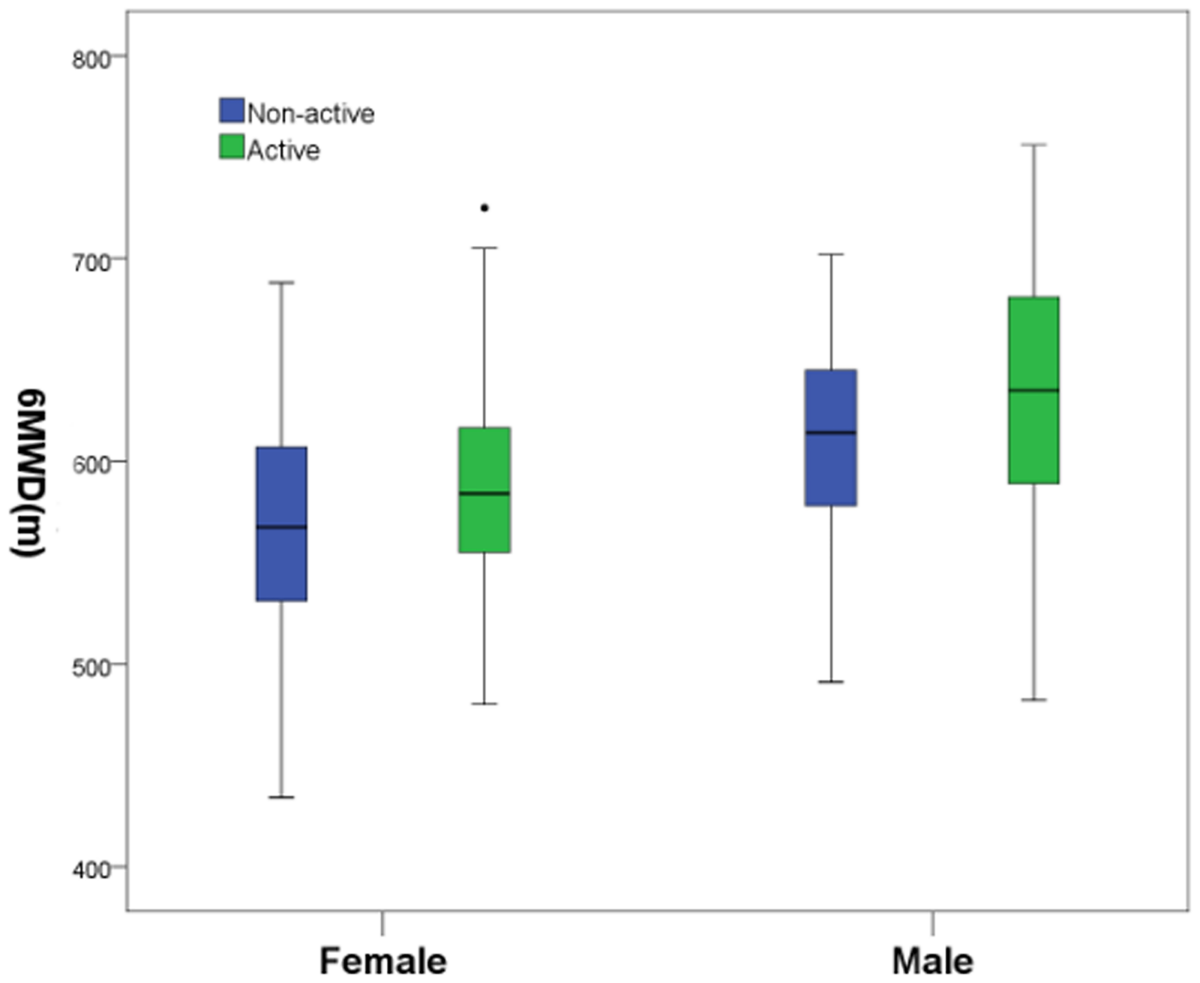
The effect of activity on the 6MWD in females and males.

**Fig 2:**
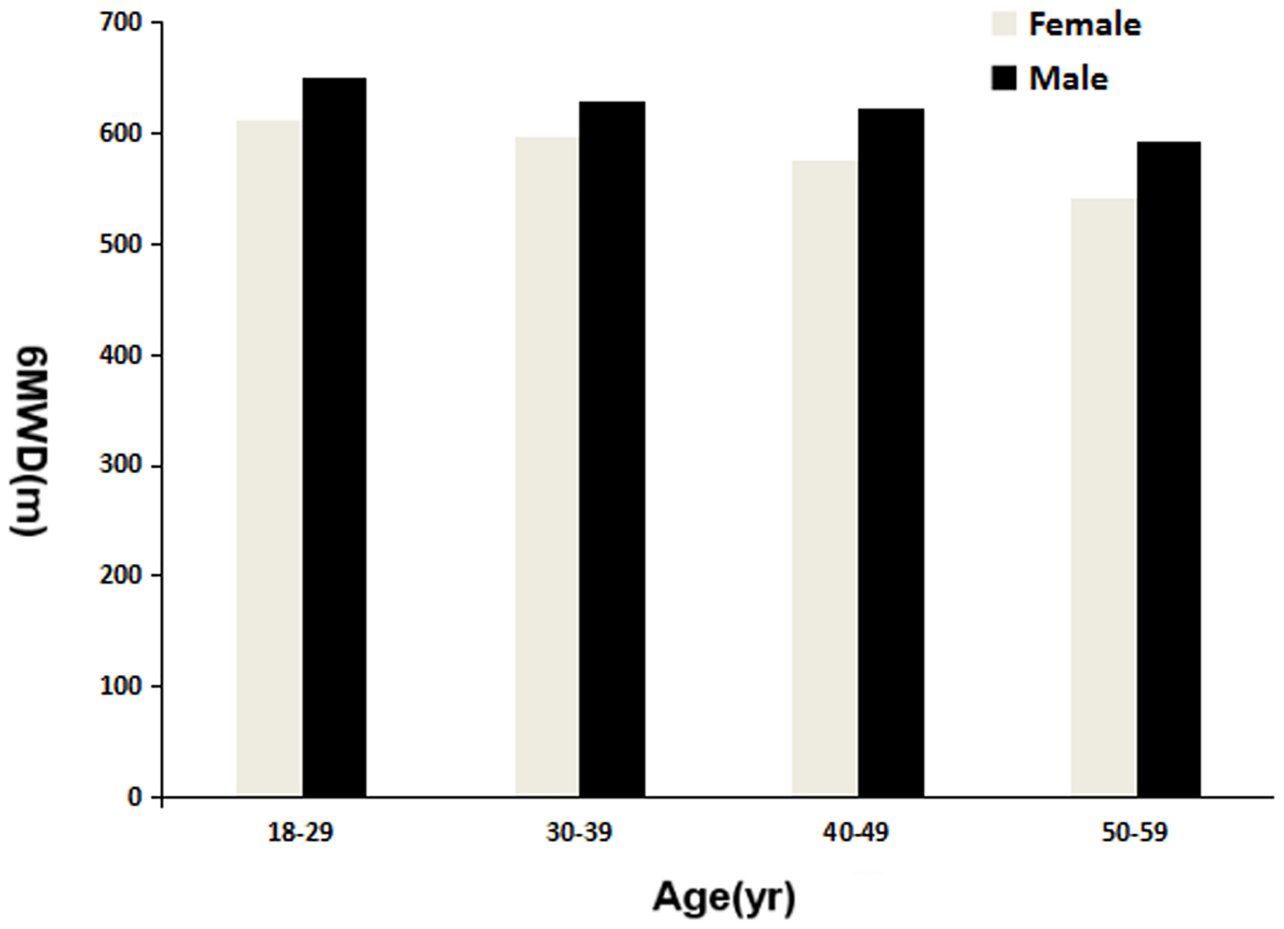
The effect of gender and age on the 6MWD of healthy subjects.

The 6MWT data for all the subjects are shown in Table 2. The mean 6MWD was 601.6±55.51 m (range, 444–756 m) for the total group, 592±53.23 m for the sedentary subjects and 609.3±56.17 m for the physically active subjects; the sedentary subjects walked significantly shorter distances than the physically active subjects (p<0.001). Fig 1 shows the effect of activity level on the 6MWD of the female and male groups. The mean 6MWD was 578±49.85 m (range, 444–725 m) for the female group and 623±52.53 m (range, 483–756 m) for the male group. The females walked significantly shorter distances than the males (p<0.05). Fig 2 shows the effect of gender and age on the 6MWD in the healthy subjects. The female and male subjects reached 62% and 60% of their mHRs, respectively, at the end of the test. We found a significant difference between the female group and the male group in terms of blood pressure, heart rate and oxygen saturation before and after the test. We did not observe clinically significant differences in the Borg values, change in oxygen saturation and heart rate before and after the test.

**Table 2:** 6MWT results for the study subjects.

Characteristic	Females (n = 319)	Males (n = 324)	p-value*	Total (n = 643)
Physically active	585.3±47.53	632.8±54.11	<0.001	609.3±56.17
Sedentary	573.3±52.02	610.5±47.81	<0.001	592±53.23
Resting HR, bpm	76.4±7.16	74.7±8.13	<0.001	75.5±7.70
Resting SpO_2_, %	98.7±0.74	97.6±0.95	<0.001	98.1±1.01
Resting systolic BP	119.8±11.12	123.2±11.09	0.004	121.5±11.23
Resting diastolic BP	74.1±7.88	76.2±7.79	0.04	75.2±7.90
6MWD, m	578±49.85	623±52.53	<0.001	601.6±55.51
Borg after 6MWT	2.3±1.0	2.2±1.5	NS	2.3±1.3
HR after 6MWT, bpm	112.0±40.82	107.4±17.30	0.021	109.7±31.32
% mHR after 6MWT	62±21.9	60±9.33	0.018	61±16.8
Difference in HR, bpm	35.4±39.75	32.7±15.17	NS	34.2±30.24
SpO_2_ after 6MWT, %	98.0±1.16	97.1±1.15	<0.001	97.5±1.24
Change in SpO_2_, %	-0.71±0.92	-0.55±0.77	NS	-0.63±0.85
Systolic BP after 6MWT	136.8±14.12	141.4±13.60	0.003	139.1±14.04
Diastolic BP after 6MWT	82.2±8.02	85.8±8.85	<0.001	84.0±8. 63

Values are expressed as the mean ± SD.

*p-value between males and females.

6MWT (D): six-minute walking test (distance); HR: heart rate; SpO_2_: oxygen saturation; % mHR: percentage of the predicted maximum heart rate; BP: blood pressure.

### Associations with the six-minute walk distance

**Fig 3:**
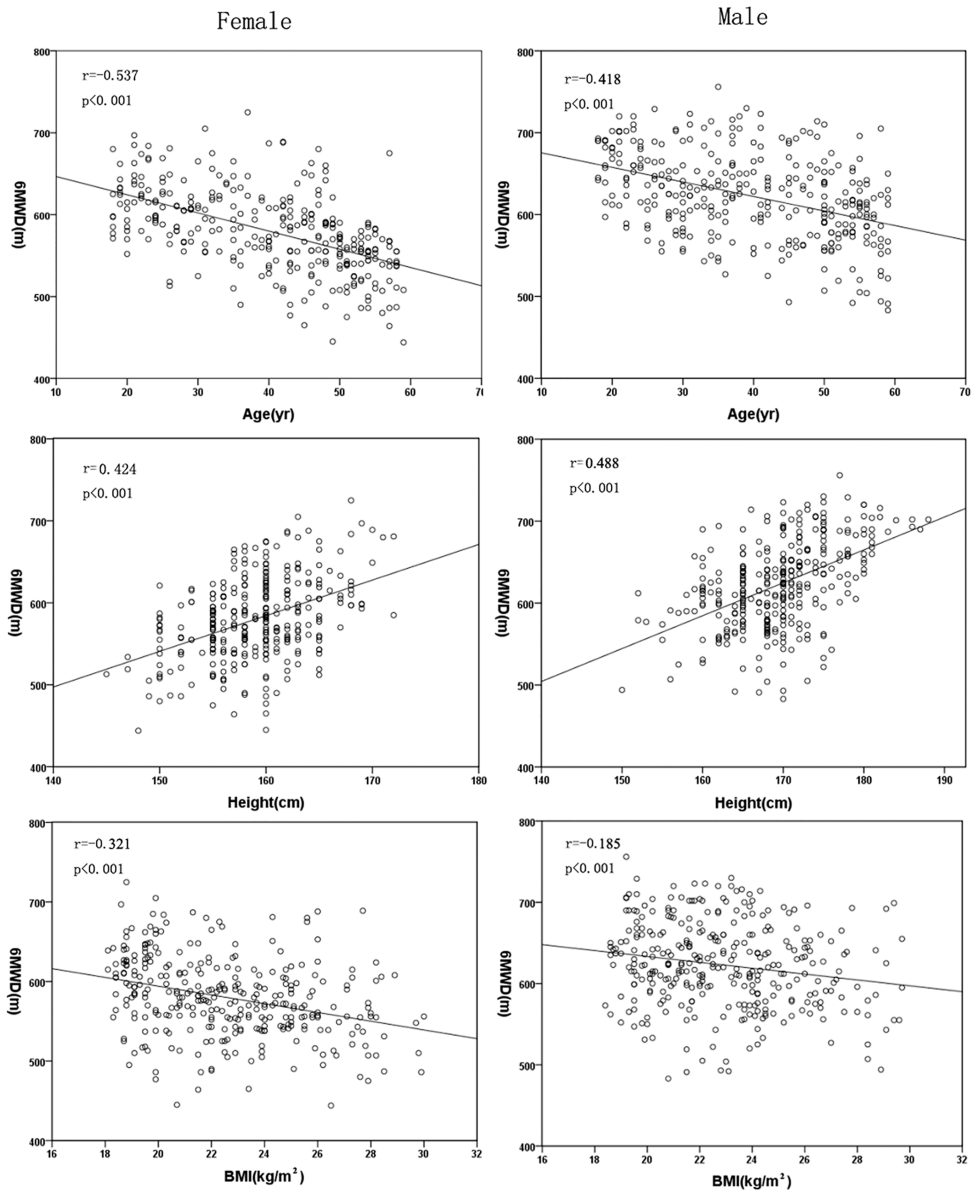
The relationship between the 6MWD and age, height and BMI for females and males.

According to the Pearson correlation, the 6MWD was significantly correlated with many demographic features and with the 6MWT variables presented in Table 3 (age, height, weight, BMI, heart rate after the test, and difference in heart rate before and after the test). Fig 3 shows the relationship between the 6MWD and age, height and BMI in the female and male groups. The variables (age, height, weight and BMI) were used in the stepwise multiple regression analysis. We found that age and height were the most significant predictors of the distance, and they explained 34% and 28% of the variance in distance for the female and male groups, respectively (Table 4).

**Table 3:** Univariate correlation coefficients for 6MWD.

Variable	Females (n = 319)		Males (n = 324)	
	r value	p-value	r value	p-value
Age	-0.537	<0.001	-0.418	<0.001
Height	0.424	<0.001	0.488	<0.001
Weight	-0.122	0.03	0.119	NS
BMI	-0.321	<0.001	-0.185	<0.001
Resting HR	-0.054	NS	-0.008	NS
HR after 6MWT	0.206	<0.001	0.358	<0.001
Difference in HR	0.220	<0.001	0.412	<0.001
Resting SpO_2_	0.026	NS	0.052	NS
SpO_2_ after 6MWT	-0.003	NS	0.047	NS
Change in SpO_2_	0.025	NS	-0.006	NS
Resting systolic BP	-0.176	0.002	-0.167	0.001
Resting diastolic BP	-0.179	0.001	-0.045	NS
Systolic BP after 6MWT	-0.243	<0.001	0.028	NS
Diastolic BP after 6MWT	-0.156	0.005	-0.016	NS

6MWD: six-minute walking distance; r value: Pearson’s correlation coefficient; BMI: body mass index.

**Table 4:** Stepwise multiple linear regression analysis, by sex, for factors associated with the 6MWD.

	Females			Males		
	Unstandardized coefficient	SE	p-value	Unstandardized coefficient	SE	p-value
Constant	233.994	83.74	0.006	141.327	78.778	0.074
Age	-1.815	0.204	<0.001	-1.039	0.226	<0.001
Height, cm	2.632	0.505	<0.001	3.083	0.438	<0.001
R^2^	0.345			0.285		
Change in R^2^	0.34			0.281		

Both height and the difference in heart rate before and after the walk test were significantly associated with the 6MWD.

The reference equations for the 6MWD are as follows:

$$Female:\;6MWD\left(m\right)=233.994-\left[age\left(yr\right)\times 1.815\right]+\left[height\left(cm\right)\times 2.632\right];r^{2}=0.34$$

$$Male:\;6MWD\left(m\right)=141.327-\left[age\left(yr\right)\times 1.039\right]+\left[height\left(cm\right)\times 3.038\right];r^{2}=0.281$$

### Comparison with published regression equations

Comparisons between the measured 6MWD values for our subjects and the predicted 6MWD values for the same age ranges using previously published reference equations from South American [11, 17], African [18], white [10, 19–21] and Asian [7, 8, 22, 23] populations are shown in Table 5. The reference equations from Chilean, white, and African populations tended to overestimate the walking distance of our subjects; Osses et al. [17] by 69.5±50.28 m (p<0.05), Ben Saad et al. [18] by 101.9±78.02 m (p<0.05), Troosters et al. [19] by 52.1±54.17 m (p<0.05), Gibbons et al. [10] by 108.1±49.13 m (p<0.05), Camarri et al. [20] by 52.08±54.17 m (p<0.05), and Jenkins et al. [21] by 119.4±50.12 m (p<0.05). By contrast, the Brazilian and the majority of Asian reference equations tended to underestimate the distance: Iwama et al. [11] by 22.2±46.10 m (p<0.05), Poh et al. [8] by 31.32±132.92 m (p<0.05), Alameri et al. [22] by 149.2±47.62 m (p<0.05), and Fernandes et al. [23] 109.1±46.72 m (p<0.05). There was no significant difference in the walking distance predicted by Korean equations and those found in the current study; Kim et al.’s [7] equation differed from the present study by 1.8±46.48 m (p>0.05).

**Table 5:** Measured 6MWD and predicted 6MWD for the same age range based on the equations reported in previous studies.

Study	Measured (m)	Predicted (m)	Measured-predicted (m)
Iwama et al.[11]	601.6±55.51	579.4±38.62	22.2±46.10*
Osses et al.[17]	599.9±55.54	669.4±56.01	-69.5±50.28*
Ben Saad et al. [18]	580.7±53.31	682.6±89.29	-101.9±78.02*
Troosters et al. [19]	569.2±48.32	621.3±30.99	-52.1±54.17*
Gibbons et al.[10]	599.9±55.54	706.9±50.33	-108.1±49.13*
Camarri et al. [20]	569.2±48.32	621.3±30.99	-52.08±54.17*
Jenkins et al. [21]	577.2±52.56	696.6±50.04	-119.4±50.12*
Poh et al.[8]	577.2±52.56	545.8±130.02	31.32±132.92*
Alameri et al.[22]	613.2±52.76	464.0±20.82	149.2±47.62*
Kim et al.[7]	597.6±55.01	595.9±22.50	1.8±46.48
Fernandes et al.[23]	594.3±54.64	485.3±31.51	109.1±46.72*

*p<0.05 according Student’s t-test.

6MWD: six-minute walking distance.

## Discussion

To the best of our knowledge, this is the first study to describe the 6MWD in the healthy Chinese Han people aged 18–59 years.

There was a significant difference in the distance walked between the female and male groups in our study (Fig 2). The male group walked a greater distance than the female group, possibly because the males were taller and had higher levels of physical activity and a greater muscle mass. There was a significant difference in the distance walked between the physically active subjects and the sedentary subjects in our study (Fig 1). Exercise physiology studies have shown that physical exercise has a significant positive correlation with muscle strength [24]. Conversely, a sedentary lifestyle usually alters the muscle metabolism, muscle mass and physical capacity [24], which could explain why the average distance walked by the sedentary subjects was significantly shorter than the distance walked by the physically active subjects in our study.

We found that age was negatively correlated with the distance walked and was the predominant variable in the regression equation for our subjects. This could be explained by the fact that muscle mass, muscle strength and maximal oxygen uptake gradually decreased with age. We observed that height was strongly correlated with the distance walked and was also the predominant variable in the regression equation for our subjects. This may be because taller height is associated with a longer stride, which makes walking more efficient, and probably results in a longer distance walked. We also found that weight and BMI were significantly correlated with the 6MWD in female subjects, but only the correlation with the BMI was observed in male subjects. BMI and weight were not represented in the final regression equation, possibly because overweight and underweight participants were excluded from our study.

In this study, we observed that the heart rate after the test and the difference in heart rate before and after the test were significantly positively correlated with the distance walked. This may be because the heart rate after the test and the difference in the heart rate before and after the test represent the level of effort the subject expended while performing the test. We found that both the resting heart rate and the heart rate after the test were significantly higher in the female subjects compared with the male subjects in our study. Previous studies have shown that heart rate differences are related to gender and that females have a higher resting heart rate than males [25, 26]. The baroreflex heart rate regulation may differ between females and males, and estrogen may affect baroreflex heart rate regulation in humans [27, 28].

The mean 6MWD in our study was 601.6±55.51 m. The forward stepwise multiple linear regression for the female and male groups showed that age and height were the most significant predictors of the 6MWD, and the regression equations could explain approximately 34% and 28% of the variance in distance for females and males, respectively.

Reference equations from Chilean [17], white [10, 19–21], and African [18] populations tended to overestimate the walking distance in our subjects. The Korean [7] estimate yielded results similar to the measured results for our population, while the reference equations from Brazilian [11], Arabian [22], Singaporean [8] and Indian [23] populations tended to underestimate the distance of our study population. These differences are commonly caused by differences in the anthropometric factors, demographic characteristics, and racial backgrounds of the recruited subjects and the standardization methods used in each study. The subjects in our study were slightly shorter than white populations, and the research was standardized according to the ATS guidelines [1]. For example, the 6MWT was performed twice and the longer 6MWD was used for further analysis in our study. The guidelines include some technical requirements to ensure the execution of the 6MWT, particularly the 30-meter length of the corridor and the inclusion of one practice walk. However, some studies did not consider these technical aspects of the 6MWT. Table 6 shows the standardization of the 6MWT used in previous studies. A longer corridor requires that subjects spend less time reversing directions, and a training effect may occur as a result of improved coordination, finding the optimal stride length, and overcoming anxiety, resulting in a greater 6MWD [29]. Three studies were consistent with our protocol. Our subjects reached an average of 61% of their mHRs, and the subjects in the study by Osses et al [17], reached an average of 74% of their mHRs. As a result, their average 6MWD values were significantly longer than those in our study. Although the subjects in the study by Iwama et al [11], reached 65 ± 13% of their mHRs, they had a greater body weight than the subjects in our study. There were no significant differences in the walking distance between the Korean [7] study and the current study, which may be due to the minimal differences in the subjects’ anthropometric factors, demographic characteristics, and racial background. In addition to the subjects' levels of daily physical activity, their attitudes and psychological factors may also influence the 6MWD [30].

**Table 6:** Standardization of the six-minute walk test used in previous studies.

Study	Track	N. of tests
Iwama et al.[11]	30 m	Two
Osses et al.[17]	30 m	Two
Ben Saad et al. [18]	40 m	Two
Troosters et al.[19]	50 m	Two
Gibbons et al.[10]	20 m	Four
Camarri et al. [20]	45 m	Three
Jenkins et al. [21]	30 m	One
Poh et al.[8]	45 m	Three
Alameri et al.[22]	30 m	One
Kim et al.[7]	30 m	Two
Fernandes et al.[23]	30 m	One
Present study	30 m	Two

Our research has some limitations. First, we did not recruit individuals who were older than 59 years of age. The 6MWT has clinical utility for several diseases, such as cardio-respiratory disease, which often occurs in the elderly population, and our reference equations are not applicable to this population. Second, in the current study, the subjects were medical personnel and workers at a public hospital, students and teachers at a local university and employees of two local private companies. They may not be representative of the entire Chinese population. Third, we did not recruit overweight people and our reference equations are not applicable to the subjects with BMI > 30 kg/m^2^. A large and multicenter study is needed to address these limitations.

In summary, age and height were the most significant predictors of the 6MWD and the regression equations explained approximately 34% and 28% of the variance in the distance for the females and males, respectively.

## Supporting information

S1 Dataset Subjects’ characteristics and 6MWT outcomes. (XLSX)
